# Effects of High Intensity Interval Training and Strength Training on Metabolic, Cardiovascular and Hormonal Outcomes in Women with Polycystic Ovary Syndrome: A Pilot Study

**DOI:** 10.1371/journal.pone.0138793

**Published:** 2015-09-25

**Authors:** Ida Almenning, Astrid Rieber-Mohn, Kari Margrethe Lundgren, Tone Shetelig Løvvik, Kirsti Krohn Garnæs, Trine Moholdt

**Affiliations:** 1 Department of Circulation and Medical Imaging, Faculty of Medicine, Norwegian University of Science and Technology, Trondheim, Norway; 2 Department of Obstetrics and Gynecology, St. Olav’s Hospital, Trondheim, Norway; 3 Department of Laboratory Medicine, Children’s and Women’s Health, Norwegian University of Science and Technology, Trondheim, Norway; VU University Medical Center, NETHERLANDS

## Abstract

**Background:**

Polycystic ovary syndrome is a common endocrinopathy in reproductive-age women, and associates with insulin resistance. Exercise is advocated in this disorder, but little knowledge exists on the optimal exercise regimes. We assessed the effects of high intensity interval training and strength training on metabolic, cardiovascular, and hormonal outcomes in women with polycystic ovary syndrome.

**Materials and Methods:**

Three-arm parallel randomized controlled trial. Thirty-one women with polycystic ovary syndrome (age 27.2 ± 5.5 years; body mass index 26.7 ± 6.0 kg/m^2^) were randomly assigned to high intensity interval training, strength training, or a control group. The exercise groups exercised three times weekly for 10 weeks.

**Results:**

The main outcome measure was change in homeostatic assessment of insulin resistance (HOMA-IR). HOMA-IR improved significantly only after high intensity interval training, by -0.83 (95% confidence interval [CI], -1.45, -0.20), equal to 17%, with between-group difference (p = 0.014). After high intensity interval training, high-density lipoprotein cholesterol increased by 0.2 (95% CI, 0.02, 0.5) mmol/L, with between group difference (p = 0.04). Endothelial function, measured as flow-mediated dilatation of the brachial artery, increased significantly after high intensity interval training, by 2.0 (95% CI, 0.1, 4.0) %, between-group difference (p = 0.08). Fat percentage decreased significantly after both exercise regimes, without changes in body weight. After strength training, anti-Müllarian hormone was significantly reduced, by -14.8 (95% CI, -21.2, -8.4) pmol/L, between-group difference (p = 0.04). There were no significant changes in high-sensitivity C-reactive protein, adiponectin or leptin in any group.

**Conclusions:**

High intensity interval training for ten weeks improved insulin resistance, without weight loss, in women with polycystic ovary syndrome. Body composition improved significantly after both strength training and high intensity interval training. This pilot study indicates that exercise training can improve the cardiometabolic profile in polycystic ovary syndrome in the absence of weight loss.

**Trial Registration:**

ClinicalTrial.gov NCT01919281

## Introduction

Polycystic ovary syndrome (PCOS) is the most common endocrinopathy in reproductive-age women and recognized as the leading cause of anovulatory infertility. The prevalence of PCOS is estimated to be up to 20% using the Rotterdam criteria [1]. The manifestations of the syndrome include hyperandrogenism, anovalutory menstrual dysfunction, and polycystic ovaries. In addition to concerns about fertility and hyperandrogenism, PCOS is also recognized as a metabolic disorder as these women have increased prevalence of insulin resistance (IR) and hyperinsulineamia, dyslipidemia, and low-grade inflammation [2–4]. The prevalence of obesity is also higher in PCOS women, and even lean women with PCOS frequently show excessive body fat and central adiposity [5, 6]. This symptom clustering shows a clear similarity to the metabolic syndrome [7]. Further, endothelial function measured using flow-mediated dilation is found to be compromised in PCOS, also after adjusting for age and body mass [8]. Taken together, this suggests that women with PCOS are at increased risk of cardiovascular disease, although study findings are equivocal [9, 10]. Weight-independent (intrinsic) IR seen in PCOS is strongly implicated in the aetiology of the syndrome, contributing significantly to the reproductive and metabolic complications of the disorder [11]. The central role of IR in the manifestations of PCOS has led to it becoming a primary target for PCOS management.

Lifestyle intervention is regarded as first-line therapy in women with PCOS [12], and has been found to improve both metabolic and reproductive manifestations of the syndrome [13]. Despite the potential beneficial effects of exercise in PCOS, there is a gap in knowledge about exercise type and intensity required to improve outcomes in this population [14]. In men at risk for IR, high intensity interval training (HIT) has been shown to have greater impact on IR than moderate continuous training [15]. Further, strength training (ST) has been found to improve insulin sensitivity in overweight/obese, sedentary men [16].

To our knowledge, no prior randomized controlled trial exists on the effects of HIT or ST as isolated interventions in women with PCOS. Our primary hypothesis was that IR would be improved after both HIT and ST. Secondary, we hypothesized that body composition, vascular function and blood lipids would improve and reproduction-related hormones normalize after both exercise regimes. Our primary objective was therefore to assess the effects of ten weeks structured exercise training on insulin sensitivity, measured with homeostatic model assessment of insulin resistance (HOMA-IR) in women with PCOS. The comparison of HIT versus ST for these hypothesized effects was exploratory.

## Materials and Methods

This pilot study was carried out at the Department of Circulation and Medical Imaging, Faculty of Medicine, Norwegian University of Science and Technology (NTNU), in collaboration with the Department of Gynecology and Obstetrics, St. Olav’s hospital, both Trondheim, Norway. The Regional Committee for Medical and Health Research Ethics in Central Norway approved the study (REK-midt 2013/886) and participants gave their written informed consent before entering.

**Fig 1:**
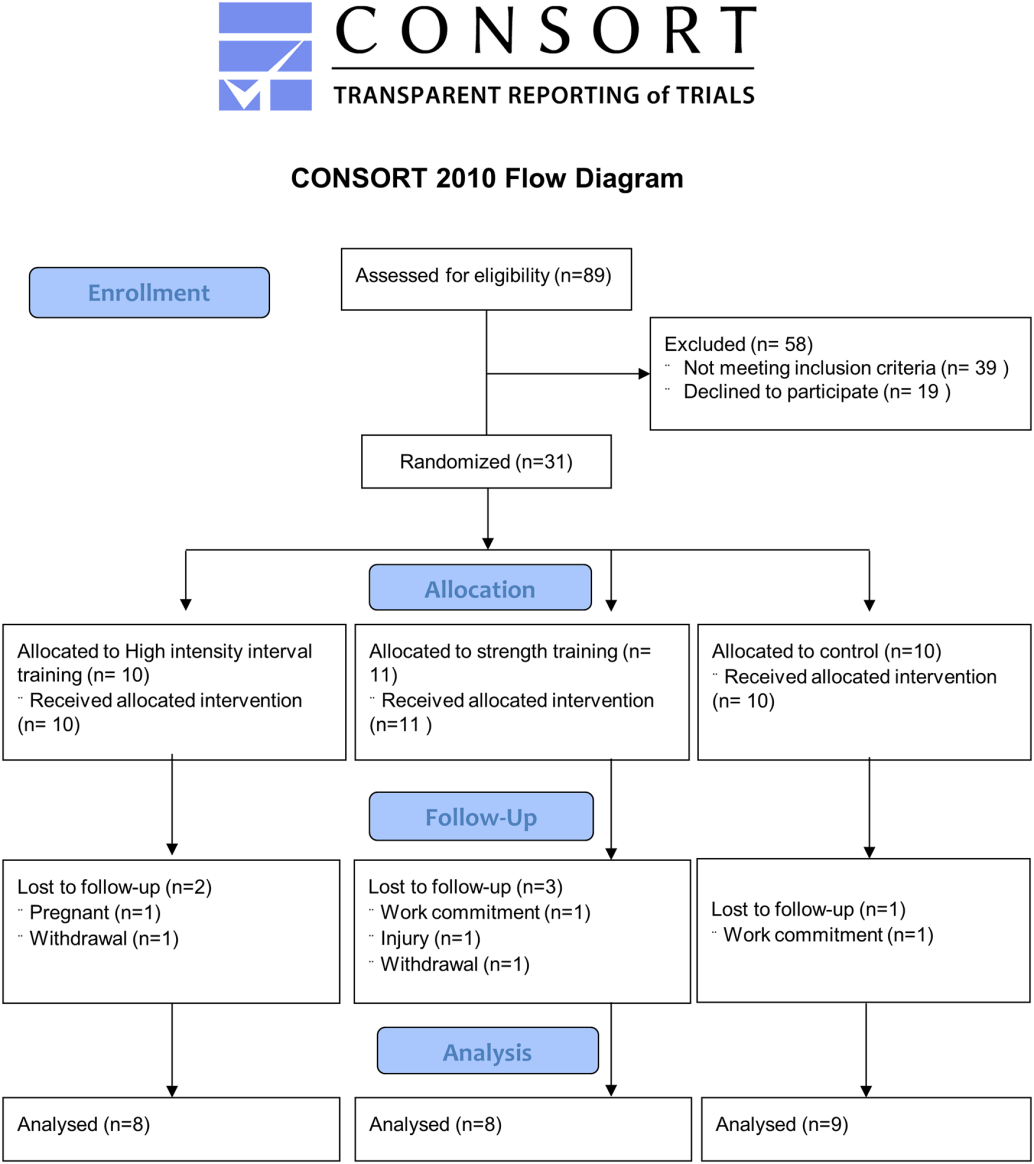
Flow-chart of the study participants.

We enrolled women with PCOS from July to October 2013 through community advertisement (Fig 1). PCOS was defined according to the Rotterdam criteria [1]: A minimum of two of the following criteria; (i) PCO morphology (12 or more 2–9 mm follicles or > 10 ml in volume, in at least one ovary), (ii) hyperandrogenism (either clinical signs as hirsutism or acne, or biochemical), and (iii) oligo/amenorrhea. Hirsutism was defined as a Ferriman Gallwey score ≥ 8 [17]. Cut-off values for biochemical hyperandrogensim was defined as testosterone >3.0 nmol/L, calculated free testosterone > 32 nmmol/L, SHBG <30 nmol/L, or free androgen index (FAI as 100 x testosterone concentration (nmol/L) /SHBG concentration (nmol/L) >5% [18]. Oligomenorrhea was defined as an intermenstrual interval > 35 days and < 8 menstrual bleedings in the past year. Amenorrhea was defined as absent menstrual bleeding or none bleeding in the past 90 days. Some of the included women were diagnosed by their own gynecologist. In women who had no prior PCOS diagnosis, we first assessed if they had oligo/amenorrhea and hyperandrogenism. If they fulfilled only one of these criteria, a vaginal ultrasound was done to confirm the diagnosis before study entry. Exclusion criteria included regular high-intensity endurance or strength training (defined as ≥ 2 sessions of vigorous exercise per week), physical ailments/injuries that limited exercise performance, on-going pregnancy, concurrent treatments (insulin sensitizers as metformin and pioglitazone) or drugs known to affect gonadotropin or ovulation, with a wash out period of one month prior to inclusion. The exception was regular use of oral contraceptives, and women were included if they did not change the type or dose > 1 month prior to the study or during the intervention period.

We calculated the study sample size based on the primary outcome measure of HOMA-IR. We selected the sample size to provide a statistical power of 80%, with a 0.05 alpha level (two-tailed), to detect a between-group difference in Δ HOMA-IR of -0.5, with a standard deviation of 0.3 [15]. The power calculation was done as a comparison between two groups (active versus control), and based on independent t-test. The comparison between the two active groups was exploratory. This gave a minimum sample size of seven participants in each group. To allow for an expected 20% lost to follow-up, we aimed at including minimum 30 participants.

Thirty-one women were stratified according to BMI (≥ or < 27) as suggested by an earlier study [19] and allocated in a 1:1:1 manner to HIT, ST or to a control group. We used a computer random number generator developed and administered at Unit for Applied Clinical Research at the University to randomize the subjects. Baseline testing was done before randomization. Follow-up testing was performed from October to December 2013, and these measurements were done non-blinded to group assignment. The subjects met in the lab after an over-night fast for ≥ 12 hours and after refraining from exercise for >72 hours at both baseline and post-intervention testing. For subjects with regular cycles, data was collected at the same day of the menstruation cycle at baseline and post-intervention testing. Which day of the menstrual cycle data was collected on was not standardized between women, but each individual was tested the same day of their menstrual cycle on baseline and post-testing.

Participants in HIT and ST were asked to attend three weekly exercise sessions for ten weeks. An exercise physiologist supervised the exercise training at least once weekly. The participants were asked to maintain their normal diet during the intervention period, without any diet plan.

**Fig 2:**
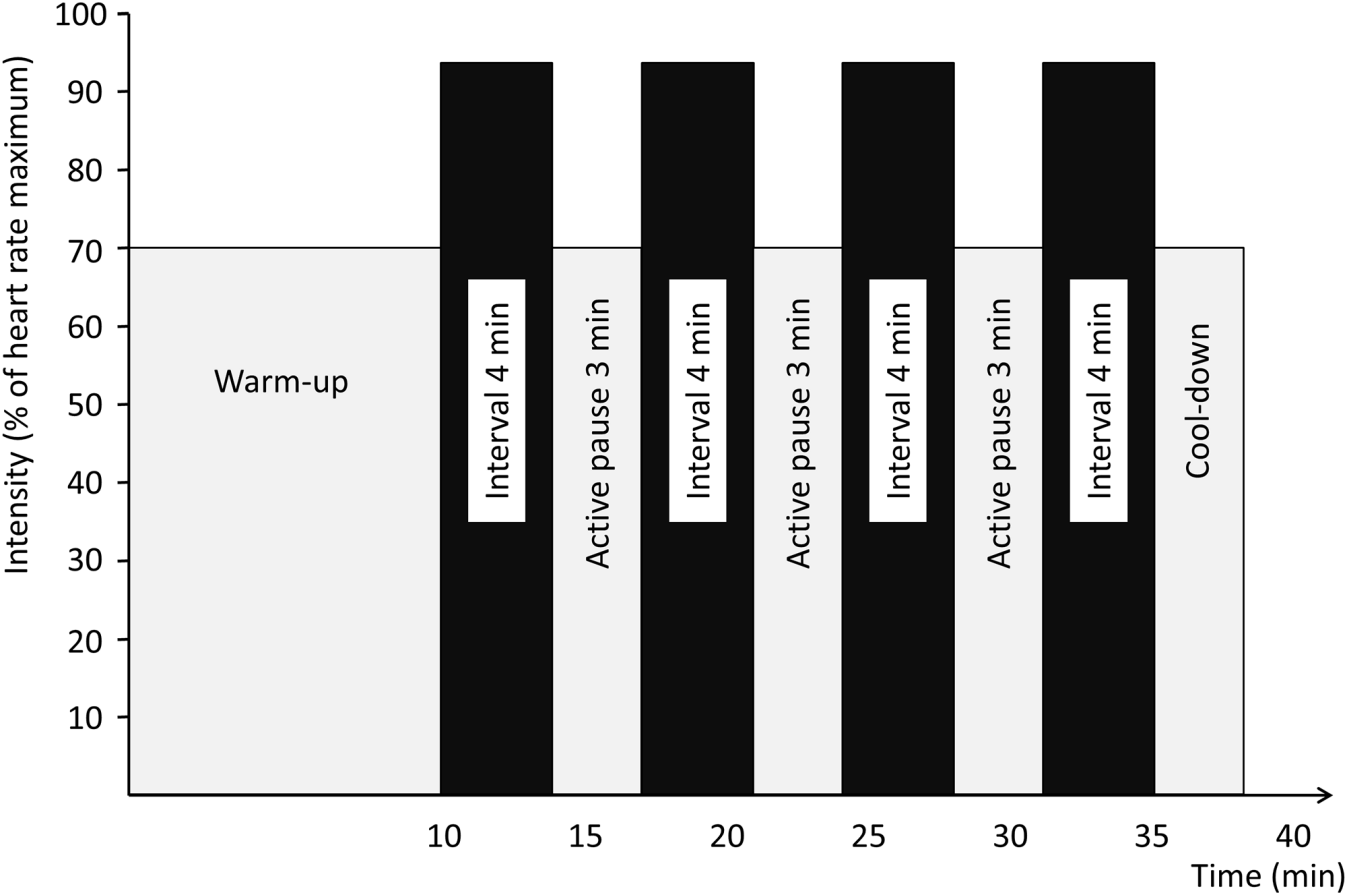
Four times four minutes high intensity interval program.

**Fig 3:**
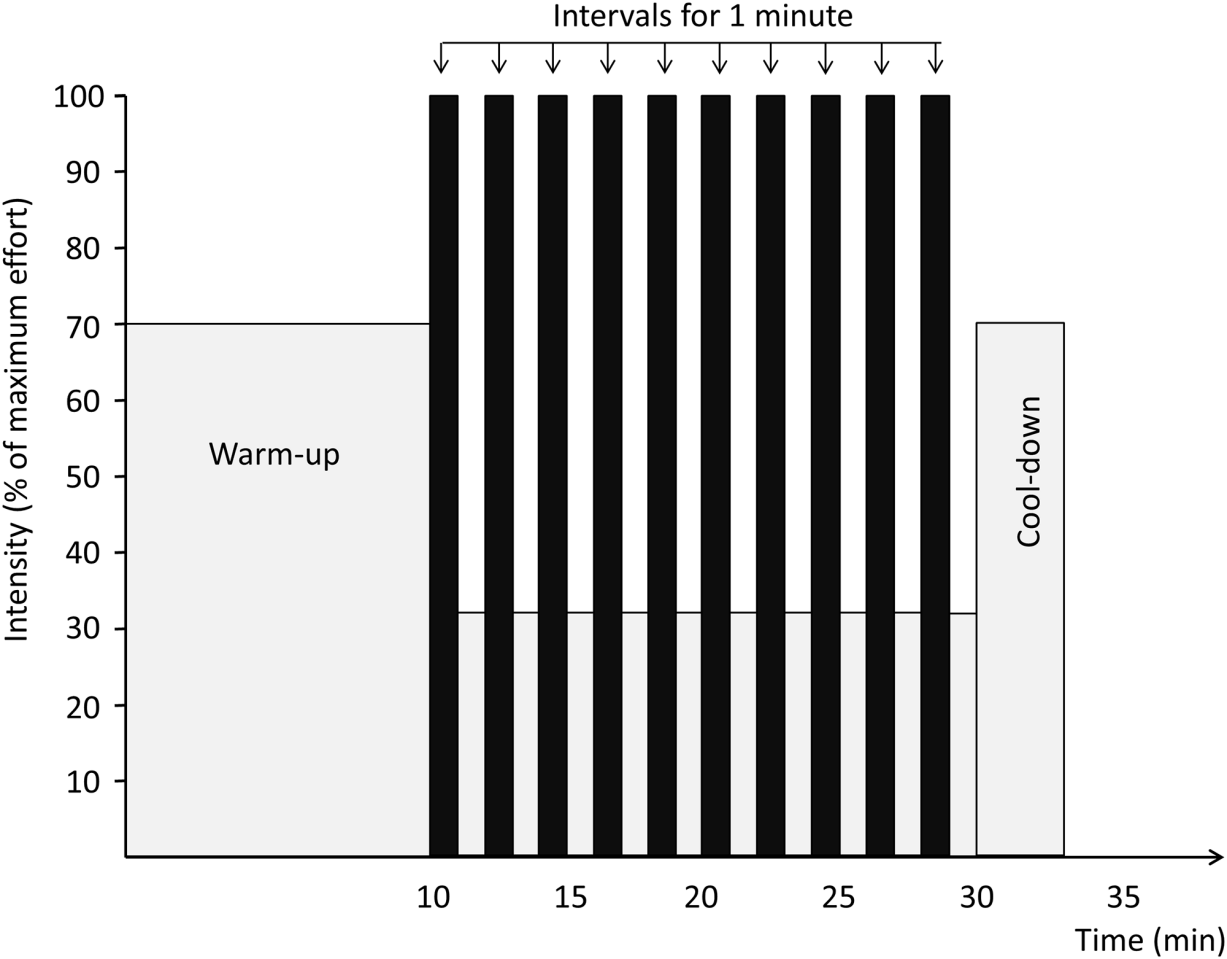
Ten times one minute high intensity interval program.

The HIT program included two weekly sessions of four times four minutes HIT at 90–95% of individual heart rate maximum (HR_max_), separated by three minutes of moderate intensity exercise at ∼ 70% of HR_max_ (Fig 2), and one weekly session of ten times one minute with maximal intensity (‘all out’) HIT, separated by one minute of rest/very low activity (Fig 3). The exercise mode was treadmill or outdoor walking/running and/or cycling (self-selected). To ensure correct exercise intensity during HIT, the subjects used heart rate monitors (Polar RCX3, POLAR, Oulu, Finland) in all sessions. Heart rate data was stored in a personal online training diary (www.polarpersonaltrainer.com) for use in analyses of compliance and exercise intensity. We checked downloaded heart rate data once weekly to ensure correct intensity.

The ST program consisted of eight dynamic strength drills with a resistance of 75% of one repetition maximum (1RM), with ten repetitions and three sets separated by one-minute rest between sets. During the intervention period, the load was increased progressively once the subjects could successfully perform three sets of ten repetitions. All sessions were conducted at a local fitness center with good access to equipment.

We advised the women in the CG to adhere to the recommended ≥150 minutes of weekly moderate-intensity exercise [20] without any follow-up during the ten weeks intervention period. After post-testing, the CG received one-month access to a local fitness center free of charge, and they were offered the HIT and ST training programs.

The primary outcome measure was change in IR from baseline to post-intervention. We calculated HOMA-IR as fasting serum insulin in μU per mL x fasting plasma glucose in mmol per L/22.5 [21]. Plasma glucose was measured using a Roche Moduclar P (Roche, Switzerland). Plasma glucose, total cholesterol, high-density lipoprotein (HDL) cholesterol, low-density lipoprotein (LDL) cholesterol, triglycerides, and high-sensitivity C-reactive protein (CRP) were measured using a Roche Moduclar P (Roche, Switzerland). Serum insulin, adiponectin and leptin were measured in duplicates by enzymed-linked immunosorbent assay (ELISA, IBL-International, Germany), using a DS2 ELISA processing system (Dynex Technologies, USA). In serum, testosterone and homocysteine were measured using mass spectrometry (Agilent 1290/Agilent 1100 HPLC analyzer, Agilent Technologies, USA), and anti-Müllarian hormone (AMH) using ELISA (STA-R Evolution, Stago, UK), SHBG and DHEAs by an immunologic method (Roche Modular E, Roche, Switzerland). We calculated free testosterone by free androgen index (FAI) as 100 x testosterone concentration (nmol/L) /SHBG concentration (nmol/L). Laboratory assays were performed in the clinical biochemistry laboratory at St.Olav’s hospital or at the Department of Circulation and Medical Imaging, Norwegian University of Science and Technology. The inter- and intra-assay coefficient variation was 2.1% and 1.5% for glucose, 2.5% and 0.9% for total cholesterol, 2.8% and 0.8% for HDL cholesterol, 2.4% and 0.8% for LDL cholesterol, 2.9% and 0.9% for triglycerides, 3.8% and <1% for high-sensitivity CRP, 5.3% and 9.5% for insulin, 7.0% and 9.9% for adiponectin, 6.9% and 8.7% for leptin, 9.3% and 5.4% for AMH, 6.0% and 3.0% for testosterone, 2.4% and 1.3% for SHBG, 4.8% and 2.1% for DHEAs, and 3.5% and 6.4% for homocysteine.

We estimated endothelial-dependent vasodilation in the left brachial artery by flow-mediated vasodilation (FMD) using the method described by Skaug et al [22]. A single experienced operator did all FMD-examinations, with use of a 12-MHz ultrasound Doppler Probe (Vivid 7 System, GE Vingmed Ultrasound, Horten, Norway). An observer blinded for group allocation analyzed the FMD data. Baseline brachial artery diameter was measured before inflation of a cuff on the forearm inflated to 250 mmHg for 5 minutes. The diameter was again measured 60 seconds after cuff release and FMD% reported is the percentage change in diameter between baseline recordings and 60 seconds after cuff release. We recorded resting heart rate as the lowest heart rate during the rest period of 5 minutes prior to FMD recordings.

Body weight, body composition and visceral fat were measured with the participants wearing light clothing and without shoes or socks using bioelectrical impedance analysis (InBody720, Biospace CO, Korea). Waist circumference was measured to the nearest 0.5 cm horizontally at the level of the umbilicus by using a metric tape with the women in standing position and at normal expiration.

Maximum oxygen uptake (VO_2max_) was measured using direct analyses of expired gas (Oxycon Pro, Jaeger, Germany). After warming up for ten minutes, the subjects walked or ran on a treadmill until exhaustion. We used an individualized ramp protocol, adjusted to last 8–12 minutes after warm-up [23]. VO_2max_ was averaged over the 30 seconds of highest oxygen uptake. As criterion for reaching that maximum exhaustion, the respiratory exchanges ratio had to be > 1.10 at the end of the test. Heart rate recovery was measured as the difference in heart rate from the maximum heart rate reached at the end of the VO_2max_ test to one minute after stopping.

Menstrual frequency was self-reported. Clinical hyperandrogenism (hirsutism) was measured by self-scoring of hair growth in seven body areas [17]. Each area was scored from 0 (absence of terminal hairs) to 4 (extensive terminal hair growth), and the scores of all areas were summed for a total hair growth score.

Observed data are reported as mean ± standard deviation (SD). We used the Kolmogorov-Smirnov test and visual inspection of histograms and Q-Q-plots to assess for normality in the data, and log-transformed when appropriate (for SHBG). We used one-way analysis of variance to compare continuous variables between groups at baseline. All subjects who met for testing after ten weeks were included in the analyses, regardless of compliance to the intervention, with outcome measures analyzed according to the treatment arm to which the subjects were randomized into. Compliance to exercise training was calculated as the number of sessions completed divided by the number of scheduled sessions in the study, in percentage. We used bivariate Pearson correlations to examine associations between HOMA-IR and body composition variables (fat percentage and visceral fat), and between HOMA-IR and VO_2max_. To assess changes in outcome variables within groups, we used univariate general linear model with the baseline value of the outcome measure as covariate. Within-group differences were considered significant when the 95% confidence interval (CI) of the estimated marginal means for each group did not include zero [24]. We used univariate general linear model covariance analysis (ANCOVA), with Bonferroni adjustment, to test differences between groups, with the difference (Δ-value) as the dependent factor, group variable as the fixed factor, and baseline values as covariates [25]. As we investigated several outcome measures, the results on secondary outcomes in this study should be regarded as exploratory. P-values of <0.05 were considered significant. We used the SPSS Statistics software program version 20 (SPSS Inc., USA).

## Results

We experienced no adverse events during the study period. Of the 31 initial subjects, six dropped out (Fig 1). Apart from lower FMD in the HIT group, there were no significant differences in baseline characteristics between groups (Table 1).

**Table 1:** Baseline characteristics of randomized participants. Variables are expressed as means ± standard deviation. P-values for between-group comparisons of continuous variables at baseline (ANOVA). BMI = Body Mass Index, VO_2max_ = Maximum oxygen uptake, HR rest = resting heart rate, HR recovery = heart rate recovery (maximum heart rate minus heart rate one minute after end of maximum effort exercise test), FMD = flow-mediated dilation, FG score = Ferriman Gallway score, OC use = oral contraceptives use, HOMA-IR = Homeostatic assessment of insulin resistance, TST = Testosterone, FAI = Free Androgen Index (Testosterone x 100/SHBG), AMH = Antimüllarian Hormone, SHBG = Sex-Hormone Binding Globulin, DHEAS = Dehydroepiandrosterone sulfate. T Chol = total cholesterol, HDL = high-density lipoprotein cholesterol, LDL = low-density lipoprotein cholesterol, TG = triglycerides.

	ST (n = 11)	HIT (n = 10)	CG (n = 10)	*p-value*
Weight, kg	76.5 ± 20.2	73.5 ± 16.7	74.5 ± 16.1	0.93
BMI, kg/m^2^	27.4 ± 6.9	26.1 ± 6.5	26.5 ±5.0	0.89
Waist, cm	94.4 ± 18.1	92.3 ± 15.8	92.9 ± 14.6	0.96
Fat mass, kg	26.7 ± 15.5	25.8 ± 12.4	26.4 ± 10.7	0.99
Fat mass, %	32.6 ± 10.4	33.2 ± 9.6	34.2 ± 6.9	0.92
Visceral fat, cm^2^	106.4 ± 54.4	103.9 ± 51.9	111,3 ± 38.7	0.94
Fat-free mass, kg	27.7 ± 3.6	26.4 ± 3.2	26.6 ± 3.6	0.63
VO_2max_, mL kg^-1^ min^-1^	38.3 ± 9.4	35.6 ± 5.6	36.1 ± 7.7	0.70
HR rest, beats/min	56.8 ± 10.0	63.4 ± 6.5	59.9 ± 12.8	0.34
HR recovery, beats	38.5 ± 10.7	33.0 ± 13.4	37.3 ± 13.9	0.60
FMD, %	5.7 ± 2.2	4.0 ± 1.2	6.2 ± 1.9	**0.04**
FG score	7.1 ± 3.3	5.7 ± 2.9	9.0 ± 3.3	0.09
OC use (n)	4	6	1	-
Regular cycles (n)	3	2	3	-
Oligomenorrhea (n)	6	7	6	-
Amenorrhea (n)	2	1	1	-
Glucose, mmol/L	5.0 ± 0.2	5.1 ± 0.3	5.0 ± 0.4	0.90
Insulin, μIU/mL*	14.9 ± 6.2	21.8 ± 7.1	15.8 ± 8.1	0.13
HOMA-IR*	3.3 ± 1.3	4.9 ± 1.7	3.6 ± 2.1	0.17
TST, nmol/L	1.4 ± 0.6	1.5 ± 1.0	1.3 ± 0.5	0.84
FAI	2.8 ± 1.7	1.9 ± 1.3	2.6 ± 1.3	0.37
AMH, pmol/L	63.0 ± 45.0	68.3 ± 54.0	53.3 ± 38.9	0.76
SHBG, nmol/L	60.7 ± 37.2	128.0 ± 110.4	57.6 ± 30.2	0.05
DHEAS, μmol/L	6.1 ± 3.1	7.2 ± 3.1	7.1 ± 2.8	0.66
T Chol, mmol/L	4.7 ± 0.8	4.5 ± 1.1	4.7 ± 0.6	0.86
HDL, mmol/L	1.5 ±0.5	1.5 ± 0.4	1.6 ±0.5	0.71
LDL, mmol/L	3.1 ± 1.1	2.5 ± 0.8	2.7 ± 0.7	0.38
TG, mmol/L	0.9 ± 0.4	1.2 ± 0.6	0.7 ± 0.3	0.10
Homocysteine*, μg/mL μmol/mL	8.5 ± 1.5	10.1 ± 7.2	8.8 ± 2.4	0.74
hsCRP, mg/L	2.6 ± 2.5	2.9 ± 3.2	1.7 ± 3.1	0.67
Adiponectin*, μg/mL	11.4 ± 2.7	11.3 ± 3.5	10.6 ± 2.7	0.84
Leptin*, ng/Ml	19.5 ± 15.4	16.5 ± 6.7	22.8 ± 14.1	0.60

* = Insulin, HOMA-IR, leptin, adiponectin and homocysteine were only measured in subjects with data after intervention, and data for insulin are only in n = 25.

In total, none of the women fulfilled all three diagnostic criteria. The number of women who had oligo/amenorrhea + hyperandrogenism was 4, 2 and 4, in the ST, HIT and control group, respectively. The number of women who had oligo/amenorrhea + PCO was 3, 5 and 2, in the ST, HIT and control group, respectively. The number of women who had hyperandrogenism + PCO was 2, 0 and 1, in the ST, HIT and control group, respectively. In addition, 2 women in ST, 3 women in HIT and 3 women in the control group were diagnosed by their own gynecologist prior to study entry.

Participants in ST performed 26 ± 6.5 exercise sessions, thereby giving a compliance of 87%. In HIT, participants performed 27 ± 1.9 sessions, thereby an average exercise compliance of 90%.

**Fig 4:**
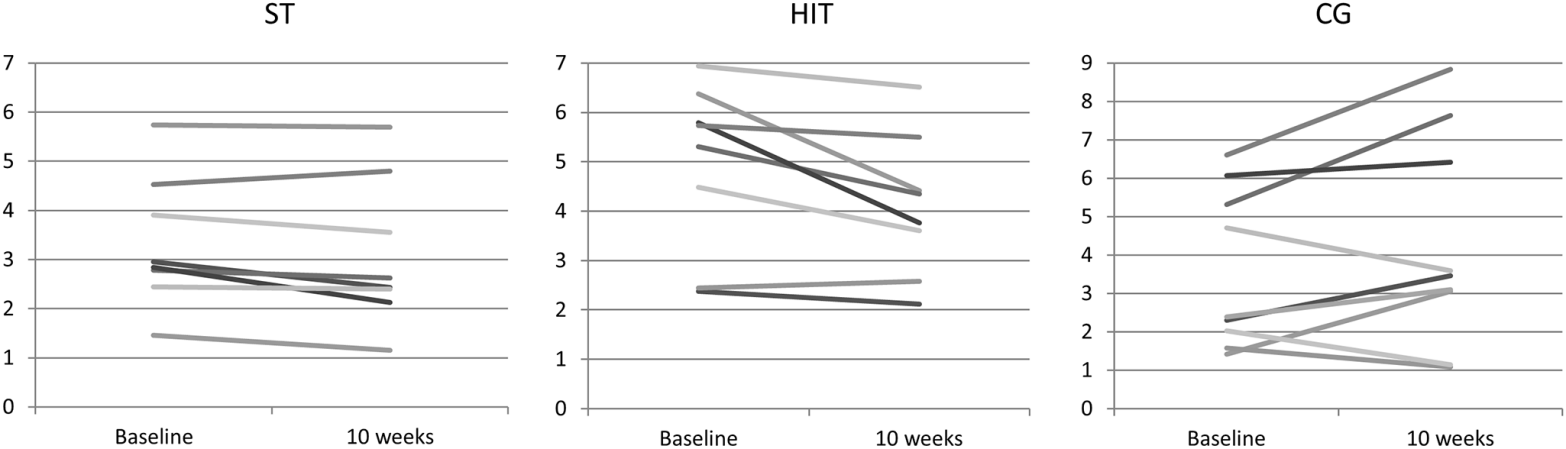
Individual changes in HOMA IR from baseline to post-intervention.

**Fig 5:**
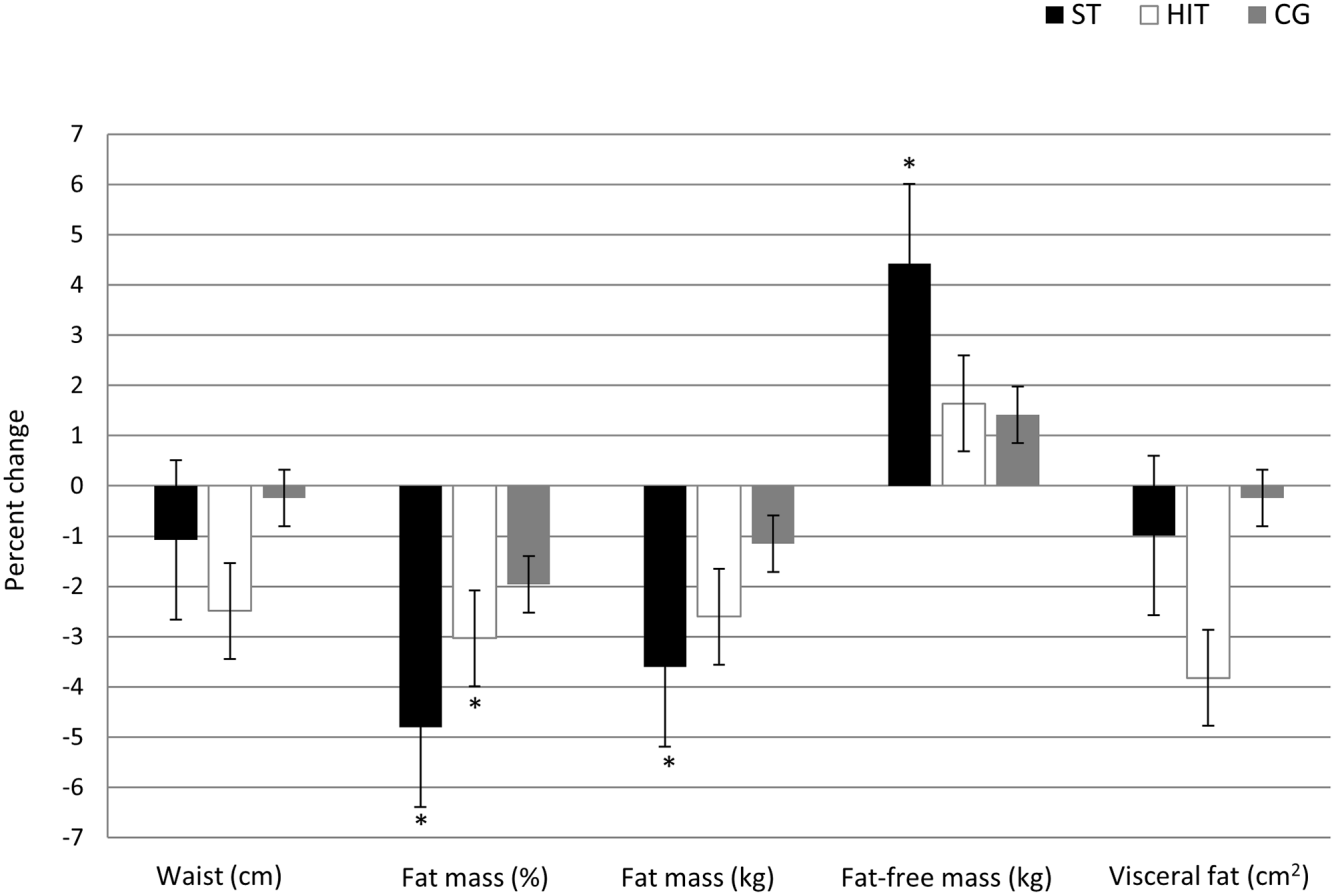
Percent change in body composition from baseline to post-intervention.

HOMA-IR improved significantly after HIT with -0.8 (95% CI: -1.5, -0.2), and with a between-group difference (p = 0.014, Table 2). Pairwise post hoc analyses showed that this difference was between the HIT and the control group only (p = 0.013). Individual changes in HOMA-IR are presented in Fig 4. After HIT, FMD increased significantly with 2.0% (95% CI: 0.1, 4.0), but with non-significant between-group difference. HDL increased significantly with 0.2 nmol/l (95% CI: 0.02, 0.5) after HIT, with a significant between-group difference (p = 0.04, Table 2). As can be seen from Table 2 and Fig 5, weight or waist circumference did not change in any group, while fat percentage decreased after both ST and HIT with -1.6 (95%CI: -2.5, -0.7) and -0.9 (95% CI: -1.8, -0.01) respectively. Fat mass in kilograms decreased after HIT with -0.6 (95% CI: -1.1, -0.004) and fat-free mass increased after ST with 1.2 (95% CI: 0.4, 2.1). There were no between-group differences in body composition outcome variables. Anti-Müllarian levels decreased after ST with -14.8 (95% CI: 21.2, -8.4), with a significant between-group difference (p = 0.04). Pairwise post hoc analyses showed that this difference was between the HIT and the ST group (p = 0.046).

**Table 2:** Outcome measures at baseline and after intervention (10 weeks) in women who completed follow-up testing. Observed values as means ± standard deviation (SD). Delta (Δ) change from baseline to 10 weeks after adjustment for baseline values, with 95% confidence interval (CI). P (values) for between-group comparisons between changes, adjusted for baseline values.

	ST (n = 8)			HIT (n = 8)			CG (n = 9)			
	Baseline	10 weeks	Δ Change	Baseline	10 weeks	Δ Change	Baseline	10 weeks	Δ Change	p
Weight, kg	77.0 ± 20.9	78.1 ± 20.0	1.1 (-1.2, 3.5)	68.3± 14.1	68.5± 14.2	0.2 (-1.0, 1.4)	75.0 ± 17.0	75.5 ± 17.5	0.4 (-1.8, 2.6)	0.66
BMI, kg/m^2^	27.1 ± 6.6	27.5 ± 6.1	0.4 (-0.3, 1.1)	23.8 ± 4.8	23.9 ± 4.8	0.1 (-0.3, 0.4)	26.3 ± 5.2	26.4 ± 5.3	0.1 (-0.7, 0.8)	0.50
Waist, cm	93.7 ± 17.6	92.3 ± 16.5	-1.4 (-3.1, 0.3)	86.8 ± 12.9	87.2 ± 12.6	0.4 (-4.4, 5.3)	92.6 ± 15.5	92.3 ± 16.4	-0.2 (-3.9, 3.5)	0.75
Fat mass, kg	27.1 ± 15.4	26.1 ± 14.7	-1.0 (-2.1, 0.1)	21.6 ± 9.4	21.0 ± 9.4	**-0.6 (-1.1, -0.004)** *	26.2 ± 11.3	25.9 ± 11.4	-0.3 (-2.2, 1.6)	0.70
Fat mass, %	33.1 ± 9.7	31.6 ± 9.4	**-1.6 (-2.5, -0.7)** *	30.2 ± 8.1	29.3 ± 8.0	**-0.9 (-1.8, -0.01)** *	33.6 ± 7.0	32.9 ± 7.3	-0.7 (-2.2, 0.9)	0.39
Visceral fat, cm^2^	106.6 ± 52.4	105.5 ± 48.4	-1.1 (-4.6, 2.5)	85.7 ± 39.3	82.4 ± 38.7	-3.3 (-6.9, 0.3)	109.9 ± 40.8	109.7 ± 41.0	-0.3 (-7.7, 7.1)	0.43
Fat-free mass, kg	27.7 ± 4.0	28.9 ± 4.1	**1.2 (0.4, 2.1)** *	25.8 ± 3.4	26.3 ± 3.2	0.4 (-0.2, 1.1)	27.0 ± 3.6	27.4 ± 3.8	0.4 (-0.3, 1.0)	0.10
VO_2max_, mL/kg/min	39.3 ± 10.2	40.2 ± 8.5	0.9 (-1.2, 2.9)	37.4 ± 4.7	41.1 ± 3.8	**3.7 (2.6, 4.8)** *	36.8 ± 7.8	36.0 ± 6.9	0.8 (-2.7, 1.0)	**<0.01**
HR rest, beats/min	55.6 ±9.8	58.3 ± 11.7	2.6 (-4.2, 9.5)	63.3 ± 7.0	59.1 ± 6.3	-4.6 (-9.6, 0.5)	59.3 ± 13.5	58.4 ± 6.2	-0.9 (-3.4, 1.6)	0.53
HR recovery, beats	35.5 ± 10.0	36.9 ± 12.1	1.4 (-9.1, 11.9)	33.9 ± 14.4	34.4 ± 8.3	0.5 (-6.4, 7.4)	36.1 ± 14.1	38.3 ± 15.2	2.2 (-10.5, 15.0)	0.85
FMD, %	5.8 ± 2.4	6.2 ± 1.4	0.4 (-1.0, 1.8)	4.0 ± 1.3	6.0 ± 1.9	2.0 (0.1, 4.0)*	6.1 ± 2.0	4.9 ± 1.9	-1.1 (-2.3, 0.03)	0.08
Glucose, mmol/L	5.1 ± 0.2	5.1 ± 0.4	0.8 (-0.15, 0.30)	5.0 ± 0.3	4.9 ± 0.2	-0.1 (-0.3, 0.1)	5.0 ± 0.4	5.0 ± 0.4	0.1 (-0.1, 0.2)	0.11
Insulin, μIU/ml	14.9 ± 6.2	13.6 ± 6.3	-1.1 (-2.6, 0.4)	21.8 ± 7.1	18.8 ± 6.7	**-3.0 (-6.0, -0.03)** *	15.8 ± 8.1	18.3 ± 11.1	2.6 (-1.6, 6.7)	**0.02**
HOMA-IR	3.3 ± 1.3	3.1 ± 1.5	-0.3 (-0.5, 0.03)	4.9 ± 1.7	4.1 ± 1.4	**-0.8 (-1.5, -0.2)** *	3.6 ± 2.1	4.3 ± 2.8	0.7 (-0.4, 1.7)	**0.01**
TST, nmol/L	1.5 ± 0.6	1.3 ± 0.5	-0.2 (-0.5, 0.1)	1.6 ± 1.1	1.6 ± 0.9	0.0 (-0.3, 0.3)	1.2 ± 0.5	1.1 ± 0.5	-0.2 (-0.5, 0.1)	0.33
FAI	2.8 ± 1.7	2.1 ± 1.1	**-0.7 (-1.3, -0.1)** *	1.5 ± 1.2	1.9 ± 1.8	0.3 (-0.6, 1.2)	2.6 ± 1.4	2.6 ± 2.5	0.0 (-1.1, 1.1)	0.28
AMH, pmol/L	48.5 ± 30.5	33.7 ± 16.5	**-14.8 (-21.2, -8.4)** *	78.5 ± 56.0	67.1 ± 31.3	-11.5 (-26.8, 3.9)	57.4 ± 38.9	52.0 ± 28.2	-5.4 (-13.2, 2.3)	**0.04**
SHBG, nmol/L	67.8 ± 41.4	82.0 ± 60.3	14.3 (-0.2, 28.7)	152.4±110.7	135.0±83.9	-17.4 (-60.3, 25.5)	56.6 ± 31.9	56.0 ± 24.7	-0.6 (-12.1, 11.0)	0.57
SHBG ln^a^	4.1 ± 0.6	4.2 ± 0.6	**0.2 (0.0, 0.3)** *	4.8 ± 0.8	4.7 ± 0.7	-0.1 (-0.4, 0.3)	3.9 ± 0.5	3.9 ± 0.5	0.0 (-0.2, 0.2)	0.39
DHEAS, μmol/L	7.4 ± 2.5	5.9 ± 2.7	-1.5 (-3.0, 0.1)	7.1 ± 3.5	5.5 ± 3.0	**-1.6 (-2.8, -0.3)** *	7.3 ± 2.8	6.3 ± 2.7	-1.0 (-2.2, 0.1)	0.71
Chol., mmol/L	4.7 ± 0.6	4.6 ± 0.4	-0.1 (-0.5, 0.2)	4.6 ± 0.9	4.5 ± 0.7	-0.01 (-0.3, 0.3)	4.5 ± 1.2	4.4 ± 1.2	-0.2 (-0.6, 1.7)	0.75
HDL, mmol/L	1.6 ± 0.5	1.6 ± 0.4	0.003 (-0.1, 0.2)	1.7 ± 0.4	2.0 ± 0.5	**0.2 (0.02, 0.5)** *	1.6 ± 0.4	1.6 ± 0.4	0.04 (-0.09, 0.2)	**0.04**
LDL, mmol/L	3.2 ± 1.1	2.6 ± 0.6	-0.6 (-1.2, 0.1)	2.3 ± 0.5	2.1 ± 0.6	-0.2 (-0.6, 0.1)	2.7 ± 1.2	2.5 ± 1.2	-0.2 (-0.6, 0.1)	0.86
TG, mmol/L	0.8 ± 0.2	0.8 ± 0.3	-0.03 (-0.3, 0.3)	1.2 ± 0.7	1.1 ± 0.7	-0.05 (-0.3, 0.3)	0.7 ± 0.2	0.7 ± 0.2	0.02 (-0.06, 0.1)	0.96
Homocys.,μmol/mL	8.5 ± 1.5	8.5 ± 1.6	0.01 (-1.0, 1.0)	10.1 ± 7.2	9.6 ± 4.0	**-0.6 (-1.0, -0.1)** *	8.8 ± 2.4	8.8 ± 4.0	-0.01 (-0.8, 0.8)	0.99
hsCRP, mg/L	1.7 ± 2.5	1.8 ± 2.7	0.04 (-0.9, 1.0)	3.2 ± 3.1	2.4 ± 2.7	-0.8 (-2.6, 1.0)	1.7 ± 1.9	1.6 ± 2.4	-0.02 (-0.6, 0.5)	0.65
Adiponect.,μg/mL	11.4 ± 2.7	11.7 ± 2.7	0.1 (-1.1, 1.3)	11.3 ± 3.5	11.4 ± 3.6	0.02 (-0.8, 0.8)	10.6 ± 2.7	11.0 ± 3.1	0.4 (-0.4, 1.2)	0.77
Leptin, ng/mL	19.5 ± 15.4	16.8 ± 10.8	-2.7 (-5.8, 0.5)	16.5 ± 6.7	15.7 ± 7.2	-0.8 (-3.9, 2.4)	22.8 ± 14.1	22.3 ± 13.2	-0.5 (-5.0, 4.0)	0.42

^a^ Variables are analysed after logarithmic transformation.

*Significant within-group changes from baseline to post-test (zero not within the 95% CI). BMI = Body mass index, VO_2max_ = Maximum oxygen uptake, HR rest = resting heart rate, HR recovery = heart rate recovery (maximum heart rate minus heart rate one minute after end of maximum effort exercise test), FMD = flow mediated dilatation, FG-score = Ferriman-Gallwey score, OC use = oral contraceptives use, HOMA-IR = Homoeostatic assessment of insulin resistance, TST = Testosterone, FAI = Free Androgen Index (Testosterone x 100/SHBG), AMH = Antimüllarian Hormone, SHBG = Sex-Hormone Binding Globulin, DHEAS = Dehydroepiandrosterone sulfat

Fat percentage was positively correlated with HOMA-IR at baseline and after ten weeks (both, r = 0.47, p = 0.02). Visceral fat was positively correlated with HOMA-IR at baseline and after ten weeks (both, r = 0.41, p = 0.04). There was no correlation between changes after the intervention period in fat percentage and HOMA-IR (r = 0.24, p = 0.25), or between changes in visceral fat and HOMA-IR (r = 0.21, p = 0.32). There was a significant inverse association between HOMA-IR and VO_2max_ at baseline (r = -0.42, p = 0.04), but not after ten weeks (r = -0.32, p = 0.12). The change in HOMA-IR correlated inversely with change in VO_2max_ (r = -0.49, p = 0.01).

## Discussion

The main findings of our study were significantly reduced IR, as well as improved endothelial function after ten weeks of HIT in women with PCOS, and improved body composition after both HIT and ST. These improvements were seen without changes in body weight. We also found indications of positive changes in reproduction-related hormonal outcomes after both HIT and ST, and improvements in HDL cholesterol and homocysteine after HIT.

This pilot study is the first randomized controlled trial to compare HIT and ST as independent interventions in women with PCOS. It is also one of few studies on effects of exercise training also including normal weight women with PCOS.

The improvement in HOMA-IR after HIT was caused by decreased fasting insulin levels, and not by change in fasting glucose. The fasting glucose levels were normal at baseline (5.0 ± 0.3 mmol/L). This is in line what the common finding of postprandial dysglycemia reported in women with PCOS, reflecting peripheral, primarily skeletal muscle, IR rather than fasting dysglycemia (for a review, see [11]). Previous randomized controlled trials on the isolated effect on IR after endurance training in women with PCOS have been using moderate intensity [26–28]. None of these studies found significantly reduced IR after exercise. Neither Sprung et al [29] found any effect of moderate intensity exercise in a non-randomized study. However, others have found improvements in IR after the combination of moderate intensity exercise and dietary intervention/advice [30, 31]. We argue that our finding of improved IR, in contrast to other trials with exercise as sole intervention, is caused by the high exercise intensity. It has been seen in other insulin resistant populations that IR is improved more by HIT compared to moderate intensity training [15, 32]. Exercise training, and in particular HIT, may improve IR by several signaling pathways, as reviewed by Gibala [33].

IR correlated with body fat percentage and amount of visceral fat, but we found no association between changes in IR and these body composition variables. This is in line with previous data [34]. We found reductions in overall body fat percentages after both ST and HIT. This was also seen in a previous study [30], whereas yet another study found no effect of exercise training on total body fat percentage [27]. These conflicting results may be due to differences between study populations at baseline, different methodology for body composition assessment, as well as different exercise regimes. We saw no significant changes in visceral fat amount after exercise, but a trend towards lower visceral fat after HIT. In contrast, Hutchinson et al [34] found reduced visceral fat after HIT in a non-controlled study on obese women with PCOS. The ST group increased their fat-free mass in parallel to the decrease in fat mass. Since skeletal muscle tissue is the primary metabolic target organ for glucose disposal, maintaining or increasing muscle mass is important in women with PCOS.

FMD has been found to be impaired in women with PCOS [8], suggesting that the risk of cardiovascular events is higher in these women. We found a significant improvement in FMD after HIT, in line with a previous study [29]. Although baseline values were included as a covariate in the analysis of FMD change, we realize that there could be a possible regression towards the mean in the HIT group. Further, the increased HDL cholesterol, the improved VO_2max_ and the reduced homocysteine levels after HIT could add benefit in regard to cardiovascular prevention. We did not, however, see any positive changes in serum markers of low-grade inflammation (adiponectin, leptin and high-sensitivity CRP).

AMH levels decreased significantly after ST. Few previous studies have assessed the effect of exercise training on AMH levels in women with PCOS, and with conflicting results. In a small non-randomized study, Moran et al [35] found decreased AMH after 12 weeks of exercise in overweight women with PCOS. In contrast, Nybacka et al [36] observed decreased AMH only after low caloric diet and not after exercise or after the combination of diet and exercise. Further, Thomson et al [37] found no significant changes in AMH levels after a weight loss intervention in overweight/obese PCOS. We are not able to offer any explanations to the discrepancy between these study results.

We did not see any significant changes in total serum testosterone after exercise training, but the ST group had significantly lower FAI after intervention due to increased SHBG levels. Some studies have found reduced testosterone and FAI after the combination of exercise and diet [30, 38], others no changes after exercise training alone [29, 31, 34]. After HIT, DHEAS was significantly reduced. The measurement of circulating DHEAS has been used as a marker of adrenal androgen secretion and excess [39]. As some of the women in our study took hormonal oral contraceptive pills (OCP), sexual hormone changes have to be interpreted with caution.

There are some limitations to our study. Our total number of participants was only 31 and when allocated to three different groups, the number of participants in each group was low. This could increase the risk for type II error. We corrected for baseline differences in our analysis to eliminate the effects that baseline differences could have on post-test values. However, the use of OCP or the fact that we did not controlled for diet may have influence some of the outcome measurements. OCP use could specifically be a potential confounder in the hormonal analyses. Studies on the pharmacological effects of OCP use on HOMA-IR are limited. However, a Cochrane review [40] on the effects of metformin and OCP in PCOS reported that fasting insulin levels and fasting glucose levels did not change with OCP treatment in any of the three trials reporting these two outcomes. In our study, women taking OCP did not change their medication type or dosage during the intervention period and none started using OCP during the study period. We also assessed a quite high number of outcome variables, without adjustments for multiple testing. This increases the risk for some of the changes seen occurring by chance. The secondary outcome variables should therefore be seen as exploratory/preliminary. Regarding the external validity of our study, we think that our population might have had an in average lower BMI than generally seen in women with PCOS. Some of the assessments were done non-blinded for group allocation. All testing at baseline was however done before randomization. The drop- out rate in our study (20%) was as expected based on experience from other clinical exercise trials, and was taken into consideration in the sample size calculation. It must be considered whether the amount of exercise prescribed in the study is feasible and realistic over time for women with PCOS who are not participating in an exercise study with close follow-up. Long-term adherence to such exercise programs is a clinically relevant issue. Further, the inclusion of two different HIT protocols for each participant within the HIT group (twice weekly 4 x 4 minutes and once weekly 10 x 1 minute) makes it difficult to isolate the effect of each exercise protocol. The women were encouraged to maintain their normal diet, but we cannot exclude the possibility for changes in diet during the intervention period, as we did not register diet throughout the study period. Insulin sensitivity was calculated with the HOMA-IR index and not the gold standard method, the euglycaemic clamp technique. HOMA-IR have some limitation and may not be as accurate as clamp in small exercise interventions [41]. However, our results are supported by other studies using both clamp and HOMA-IR to measure insulin sensitivity in exercise interventions with moderate and/or high intensity exercise [15, 32, 42].

In conclusion, this pilot study indicated that ten weeks of HIT improved IR and FMD, in women with PCOS. Both HIT and ST improved body composition. Changes were seen after exercise intervention as the sole treatment and without changes in weight. These findings may have important implications for exercise training in management and treatment of PCOS. However, further research is needed to advance our conclusions, and establish exercise guidelines for these women.

## Supporting Information

S1 CONSORT Checklist (DOCX)

S1 Protocol (DOCX)

S1 Dataset (SAV)

S2 Dataset (SAV)
