# Supplementary material for: Effects of High Intensity Interval Training and Strength Training on Metabolic, Cardiovascular and Hormonal Outcomes in Women with Polycystic Ovary Syndrome: A Pilot Study
Source: PLoS One. 2015 Sep 25;10(9):e0138793. doi: 10.1371/journal.pone.0138793 (PMC4583183; doi:10.1371/journal.pone.0138793)
Supplement: S1 Protocol — (DOCX) [file pone.0138793.s002.docx]

## High intensity interval training or strenght training in women with polycystic ovary syndrome. A randomized, controlled trial

Collaborators: Trine Moholdt (principal investigator), Ida Almenning (collaborator), Astrid Rieber-Mohn (collaborator)

Contact information: [trine.moholdt@ntnu.no](mailto:trine.moholdt@ntnu.no) 0047 97098594

# Introduction

**Background and rationale**

Polycystic ovary syndrome (PCOS) is a common endocrine disorder of women in reproductive age affecting 6-20%^[1](#_ENREF_1)^. Women with PCOS are more commonly overweight or obese (38-66%), although this does not form part of the diagnostic criteria for the syndrome. PCOS is also a major unrecognized cardiovascular disease risk factor because of the increased prevalence of subclinical atherosclerosis, type 2 diabetes, dyslipidemia and impaired glucose tolerance^[2](#_ENREF_2)^. Although not included in the diagnostic criteria, weight-independent insulin resistance is strongly implicated in the aetiology of the syndrome, as more women with PCOS have insulin resistance and type 2 diabetes compared to weight-matched controls^[3](#_ENREF_3)^. The mechanisms underlying this intrinsic insulin resistance in PCOS remains unclear, but potentially these are associated with increased abdominal visceral fat^[4](#_ENREF_4)^. In other insulin resistant populations, exercise training has consistently been shown to improve cardiovascular risk factors and reduce type 2 diabetes risk[^5^](#_ENREF_5)^,^[^6^](#_ENREF_6). Furthermore, insulin sensitivity has been found to increase after a period of exercise training[^6^](#_ENREF_6). Lifestyle intervention is regarded as first-line therapy in women with PCOS[^7^](#_ENREF_7), however, there is a lack of well-designed studies on the effect of exercise training on insulin sensitivity and reproductive outcomes [^3^](#_ENREF_3) In summary, prior trials have showed improved insulin sensitivity and reproductive function (measured as changes in menstrual function, ovulation, or pregnancy rates) after a period of exercise training. Results on other cardiovascular outcomes, including blood pressure and lipid profile, overweight/obesity and abdominal visceral fat, have been conflicting across studies. In other insulin resistance populations, both aerobic training, strength training or the combination of aerobic and strength training have been found to increase insulin sensitivity and to improve body composition[^8-11^](#_ENREF_8) No prior studies have examined the effect of strength training alone on insulin sensitivity, reproductive outcomes and body composition in women with PCOS. Most of the prior studies on aerobic exercise in PCOS have had moderate exercise intensity. We know from other insulin resistant populations that high intensity interval training is superior to moderate training in improving insulin sensitivity[^12^](#_ENREF_12)

**Research question**

Our research question is whether strength training or high intensity interval training is effective in improving insulin sensitivity, cardiovascular outcomes (peak oxygen uptake, lipid profile, endothelial function), body composition and reproductive outcomes (hormones, ovulation) in women with PCOS.

**Specific objectives or hypothesis**

The main objective of this study will be to examine the effects of structured exercise training for 10 weeks in women with PCOS. We hypothesize that both strength training and high intensity interval training will improve insulin sensitivity and body composition. A secondary hypothesis is that ovulation will improve in both the exercise training groups compared to the control group.

# Methods

**Trial design**

This is a randomized, controlled trial with three parallel groups. The allocation to this superiority trial will be 1:1:1 to a high intensity interval training (HIT) group, a strength group (ST) and a control group (CG).

**Participants**

Eligibility criteria (inclusion and exclusion criteria).

Inclusion criteria are PCOS according to the Rotterdam criteria[^13^](#_ENREF_13): two of three of hyperandogenism, irregular anovalutory periods or ultrasound polycystic ovary (PCO) morphology. Women should be 18-45 years.

Exclusion criteria includes regular high intensity endurance or strength training (two or more times per week of vigorous exercise), concurrent treatments (insulin sensitizers or drugs known to affect gonadotropin or ovulation, with a wash out period of 1 months prior to inclusion), on-going pregnancy.

**Screening, recruitment and participant timeline**

The study will be announced at the hospital and university homepages, at local stores and public places. Patients who have a PCOS diagnosis from a gynecologist will not have to go through additional screening. Other subjects will go through a screening prior to baseline testing to confirm that they have PCOS according to the Rotterdam criteria as described above. From inclusion in the study until the end of the post testing, it will be 11-13 weeks for each participant. In addition, we will do follow-up data collection regarding reproductive outcomes for 16 more weeks, making the individual participation period 27-29 weeks from inclusion (Figure 1)

**Figure 1. Flow chart of participants in the study**

**Interventions**

Subjects will be stratified according to BMI (above or below 27) and randomized to one of three groups after baseline testing, using a computer random number generator at another unit at the university.

High intensity interval aerobic exercise training (HIT)

The subjects will do high intensity interval training (HIT) three times per week. The three weekly supervised HIT sessions will include two 4x4 min interval sessions and one 10x1 min session. The HIT consist of “uphill” treadmill running/walking. At the 4x4 minute intervals, the subjects will do a 10 minute warm-up at 60 % to 70 % of maximum heart rate (HR) followed by four 4-minute intervals reaching 90 % to 95 % of individual HR_max_. Each interval will be separated by 3-minute active pauses of running/walking at 60% to 70% of max HR. The training session will be terminated by a 10-minute cool-down at 50% to 70% of max HR. Total exercise time is estimated to 45 minutes (Figure 2). At the 10x1 minute intervals, the subjects will do a 10 minute warm up at 60% to 70% of maximum heart rate (HR) before exercising ten 1-minute intervals at maximal intensity (that can be performed for one minute). Each interval will be separated by one minute of very light intensity exercise, walking at low pace. The training session will be terminated by a 5-minute cool-down at 50% to 70% of max HR. Total exercise time is estimated to 36 minutes (Figure 3).


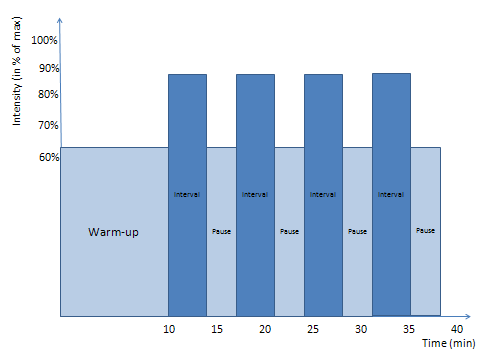


**Figure 2. High intensity interval training, 4 x 4 min model**

**
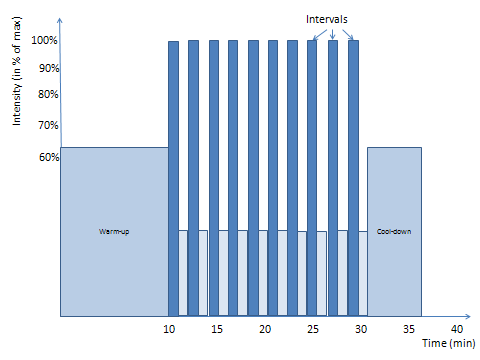
**

**Figure 3. High intensity interval training, 10 x 1 min model**

Strength training (ST)

The subjects will attend for supervised training three sessions per week for 10 weeks (9-12). The strength training program involves dynamic contraction at intensities of 60 – 70 % of 1 repetition maximum (RM) to improve strength and muscle hypertrophy. Each session will contain 8 exercises on the major muscle groups: Squats, deadlift, lunge, standing bent rowing, shoulder press, bench press, push-ups, and abdominal crunches. Each drill consists of 10-12 repetitions (reps) x 3 sets separated by one minute rest between sets. To ensure good technique, the subjects will be supervised by a trainer. It will be “rest-days” between sessions to ensure that the subjects are fully recovered. In order to progress in strength, there will be a gradual increase in resistance (kg) to maintain the number of repetitions recommended.

Equipment: dumbbells, barbell, step. Total exercise time is estimated to 45 minutes.

Control group (CG)

Based on the Norwegian recommendation we will encourage the control group to perform 60 minutes of physical activity at moderate to high intensity on a daily basis (REF: Sosial- og helsedirektoratet (2005) Norske anbefalinger for ernæring og fysisk aktivitet).

**Outcomes**

All outcome measures will be assessed at baseline and after the 10 weeks intervention period if not otherwise stated. The primary outcome of this study will be insulin sensitivity, measured with the homeostatic model assessment for insulin resistance (HOMA-IR) method. HOMA-IR is calculated as (FPI*FPG)/22.5, where FPI and FPG are fasting insulin and fasting glucose, respectively. Secondary outcomes include: body weight (in kg), BMI (in kg/m^2^), body composition (in percentage body fat, using InBody impedance scale), fat distribution (as waist circumference measured in cm at the level of the umbilicus and using InBody impedance scale). Other measures will be aerobic capacity measured as maximum oxygen uptake (in mL/min/kg), maximal leg and chest strength (in one repetition maximum in a hack lift machine and in bench press), endothelial function measured as flow-mediated dilatation of the brachial artery (in % change compared to baseline) and blood pressure (diastolic and systolic, in mmHg. In blood we will measure: lipids (total cholesterol, HDL, LDL, triglycerids, all in mmol/l), anti-mullerian hormone (AMH), and HbA1c.). We will also measure biochamical hyperandrogenism: total testosterone (in nmol/L), sex hormone binding globulin (SHBG, in nmol/L), free androgen index (FAI), and clinical hyperandrogenism (Ferriman-Gallwey score).

Ovulation frequency will be measured during the intervention period of 10 weeks and in the following 16 weeks (26-29 weeks from baseline). We will use menstruation diaries to measure this.

**Sample size**

We selected the sample size to provide a statistical power of 80%, and with a 0.05 alpha level (two-tailed), to detect a difference in HOMA-IR of 18%, based on a previous study.[^10^](#_ENREF_10) This gives a minimum sample size of seven subjects in each group. To allow for 20% drop out, and due to some uncertainty in the calculation, we will aim at including 10 subjects in each group, giving a total of 30 subjects.

**Study setting**

This study will be done in an academic hospital in Norway. This study will be a master thesis project for two master students.

**1.** Yildiz BO, Bozdag G, Yapici Z, Esinler I, Yarali H. Prevalence, phenotype and cardiometabolic risk of polycystic ovary syndrome under different diagnostic criteria. *Hum Reprod.* Oct 2012;27(10):3067-3073.

**2.** Alexander CJ, Tangchitnob EP, Lepor NE. Polycystic ovary syndrome: a major unrecognized cardiovascular risk factor in women. *Rev Obstet Gynecol.* Fall 2009;2(4):232-239.

**3.** Harrison CL, Lombard CB, Moran LJ, Teede HJ. Exercise therapy in polycystic ovary syndrome: a systematic review. *Hum Reprod Update.* Mar-Apr 2011;17(2):171-183.

**4.** Hutchison SK, Stepto NK, Harrison CL, Moran LJ, Strauss BJ, Teede HJ. Effects of exercise on insulin resistance and body composition in overweight and obese women with and without polycystic ovary syndrome. *J Clin Endocrinol Metab.* Jan 2011;96(1):E48-56.

**5.** Irving BA, Davis CK, Brock DW, et al. Effect of exercise training intensity on abdominal visceral fat and body composition. *Med Sci Sports Exerc.* Nov 2008;40(11):1863-1872.

**6.** Tjonna AE, Lee SJ, Rognmo O, et al. Aerobic interval training versus continuous moderate exercise as a treatment for the metabolic syndrome: a pilot study. *Circulation.* Jul 22 2008;118(4):346-354.

**7.** Moran LJ, Pasquali R, Teede HJ, Hoeger KM, Norman RJ. Treatment of obesity in polycystic ovary syndrome: a position statement of the Androgen Excess and Polycystic Ovary Syndrome Society. *Fertil Steril.* Dec 2009;92(6):1966-1982.

**8.** Kim ES, Im JA, Kim KC, et al. Improved insulin sensitivity and adiponectin level after exercise training in obese Korean youth. *Obesity (Silver Spring).* Dec 2007;15(12):3023-3030.

**9.** Stensvold D, Tjonna AE, Skaug EA, et al. Strength training versus aerobic interval training to modify risk factors of the metabolic syndrome. *J Appl Physiol.* Jan 21 2010.

**10.** Earnest CP, Lupo M, Thibodaux J, et al. Interval Training in Men at Risk for Insulin Resistance. *Int J Sports Med.* Nov 23 2012.

**11.** Thomson RL, Brinkworth GD, Noakes M, Clifton PM, Norman RJ, Buckley JD. The effect of diet and exercise on markers of endothelial function in overweight and obese women with polycystic ovary syndrome. *Hum Reprod.* Jul 2012;27(7):2169-2176.

**12.** Hwang CL, Wu YT, Chou CH. Effect of aerobic interval training on exercise capacity and metabolic risk factors in people with cardiometabolic disorders: a meta-analysis. *J Cardiopulm Rehabil Prev.* Nov-Dec 2011;31(6):378-385.

**13.** Revised 2003 consensus on diagnostic criteria and long-term health risks related to polycystic ovary syndrome (PCOS). *Hum Reprod.* Jan 2004;19(1):41-47.
